# The Effect of Being Vaccinated and National Vaccination Rates on Individuals’ Cognitions, Emotions, and Economic Expectations: Evidence from Israel

**DOI:** 10.1007/s12529-024-10269-3

**Published:** 2024-02-12

**Authors:** Eyal Lahav, Shosh Shahrabani, Mosi Rosenboim, Yoshiro Tsutsui

**Affiliations:** 1 The Open University of Israel, University Road 1, Raanana, Israel; 2 Economics and Management Department, The Max Stern Yezreel Valley College, Emek Yezreel, Israel; 3 Guilford Glazer Faculty of Business and Management, Ben Gurion University of the Negev, Beer Sheva, Israel; 4 Kyoto Bunkyo University, Kyoto, Japan

**Keywords:** Emotions, Vaccination, Perceived risk, Prevention behavior, COVID-19

## Abstract

**Background:**

Israel was the first nation to establish a vaccination program during the COVID-19 pandemic. Thus, its citizens could look to no other country to help them judge its influence. People’s predictions of their safety should depend on whether they were vaccinated, whereas their predictions regarding the COVID-19 pandemic should be based on the degree of progress of the vaccination program. We expected people to understand that the program would improve their safety by reducing the number of infected people around them.

**Method:**

An original panel survey was conducted covering the pandemic’s first year. Respondents reported their vaccination status, emotions, evaluations of their safety, and predictions about the spread of COVID-19 and the Israeli gross domestic product.

**Results:**

Estimates of fixed-effects models using the survey data suggest that being vaccinated affected people’s cognitions about their own safety and their emotions but not their expectations regarding the situation in Israel as a whole. In contrast, the vaccination rate in Israel affected only the respondents’ expectations about the spread of COVID-19, their own income, and Israel’s gross domestic product.

**Conclusions:**

Being vaccinated is important for people’s personal emotional and cognitive relief during a pandemic. A high vaccination rate improves people’s economic expectations, which is important to the recovery of economic activity.

**Supplementary Information:**

The online version contains supplementary material available at 10.1007/s12529-024-10269-3.

## Introduction

Vaccination is one of the few tools for combatting infectious viruses [1, 2]. Yet, studies have found refusal or hesitancy to vaccinate against COVID-19 worldwide (e.g., [3, 4]). Such hesitancy prevents a society from reaching the level of vaccination required for the social optimum. Achieving this level requires that people understand the direct effect of being vaccinated on themselves and the indirect effect of doing so for society in general. This study explores the mechanism through which vaccination contributes to society. Israel serves as a best-case study for examining this because during the COVID-19 pandemic, Israel was the first country to embark on a rapid vaccination program with a novel vaccine. Thus, its citizens could not look at other countries to see the effectiveness of vaccination at the country level.

Vaccination programs affect people individually and society in general. Becoming vaccinated changes people’s cognitions about their safety and emotions. Increased vaccination rates within a society protect everyone by reducing new infections and hospitalizations, which allows the reopening of various activities, including the economy. Therefore, as vaccination rates increase, people revise their predictions regarding future economic activity upward. This revised outlook stimulates their investment and consumption, which helps societies recover. Furthermore, the increase in the rate of vaccinated people, in turn, improves an individual’s personal safety by reducing the number of infected people around them. Many people understand these relationships. Nevertheless, throughout the pandemic, there were many people worldwide who were hesitant about becoming vaccinated or refused to do so.

### The Current Study

The aim of this study was to investigate how the COVID-19 vaccination program in Israel affected people through both their personal decision to become vaccinated and the vaccination rates nationally. An original panel survey was conducted covering the year from the onset of the COVID-19 pandemic in March 2020 to the return to everyday life in April 2021. The survey asked the respondents to report their vaccination status, emotions, evaluations of their safety, and predictions regarding the spread of COVID-19 and the gross domestic product (GDP) in Israel. This approach made it possible to investigate the mechanisms underlying the effects of the vaccination program. Fixed-effects models using a year’s worth of panel data were estimated to control all of the time-invariant variables and determine the effects of the vaccination program in Israel as a within-individual change. The focus was on determining if becoming vaccinated personally and the vaccination rate nationally had different effects.

### Literature Review

The COVID-19 pandemic heightened people’s negative emotions, such as anxiety, fear of contracting the virus, and concerns about the safety of the vaccine [5–7]. Several theories suggest that emotions play a central role in preventive health behavior. One example is the common sense model [8], which assumes that decision makers seek to regulate threats to their health and emotions [9]. Previous empirical studies have also shown that deciding to get vaccinated correlates with negative emotions. For example, Thompson et al. [10] indicated that vaccinated workers were more “worried about getting the flu” than unvaccinated workers. Other studies reported that those who felt more susceptible to getting an infection had stronger intentions of getting vaccinated [11–14]. Chapman and Coups [9] found that an anticipated reduction in worry and concern by getting vaccinated predicted the decision to do so. However, anticipated emotions do not always predict later emotional experiences accurately. The current study adds to the existing literature by examining the effect of the decision to get the novel COVID-19 vaccine on the Israeli population’s experienced emotions of fear and anxiety.

Previous studies have also shown that communicating the collective benefits of herd immunity is associated with significantly reduced vaccine hesitancy [15, 16]. However, we are unaware of studies that examined the effect of communicating the national vaccination rate on perceptions of herd immunity or, in other words, expectations about the future spread of COVID-19. The current study fills this void, because in Israel vaccination rates and the concept of herd immunity received comprehensive media coverage from government representatives and news reporters. Thus, it was possible to examine whether higher national vaccination rates are associated with expectations about containment of the future spread of COVID-19.

By June 2020, many people worldwide had experienced a decline in their incomes, and their optimism about economic recovery had not fully rebounded [17]. The current study explored if there is a link between public vaccination rates and the level of optimism about economic expectations. Benhabib and Spiegel’s [18] study in the United States revealed a positive empirical relationship between consumer expectations and the following year’s economic activity in the country. Given the declines worldwide in income and economic activity due to the pandemic, a resurgence of optimism about economic expectations could result in more positive economic decisions and expenditures, ultimately contributing to economic recovery. Therefore, it is crucial to investigate the relationship between vaccination rates and economic expectations.

The rest of this paper is organized as follows. Details on the vaccination process in Israel are provided next, followed by a description of the survey and the introduction of the questions, key variables, and estimation method. The Results and Discussion follow.

### The Vaccination Process in Israel

On November 9, 2020, Pfizer [19] announced the successful results of clinical trials of their COVID-19 vaccine. A few days later, following negotiations with the company, the Israeli government signed a contract with Pfizer to purchase vaccines for about half of its citizens.

Vaccinations began on December 20, 2020, during the third COVID-19 outbreak in Israel. According to the priorities set for the vaccines, medical staff were vaccinated first, followed by people aged 60 and over and at-risk populations. The vaccine was then made available to those who were at high risk of exposure to the virus such as teachers and daycare providers.

As soon as the vaccine became available, the four national Health Maintenance Organizations (HMOs) opened up vaccination centers throughout the country to speed up the process. Indeed, 72,000 residents were vaccinated in the first three days. After three weeks of vaccinations, 65% of those aged 60 and over were vaccinated. By January 10, 2021, vaccine eligibility was being gradually expanded, initially to those aged 50 and above, then 40, then 35. On February 4, 2021, vaccinations were offered to those aged 16 and up. Everyone in Israel must belong to one of the HMOs, and the vaccine was made available to everyone for free.

## Method

### The Survey

**Fig. 1:**
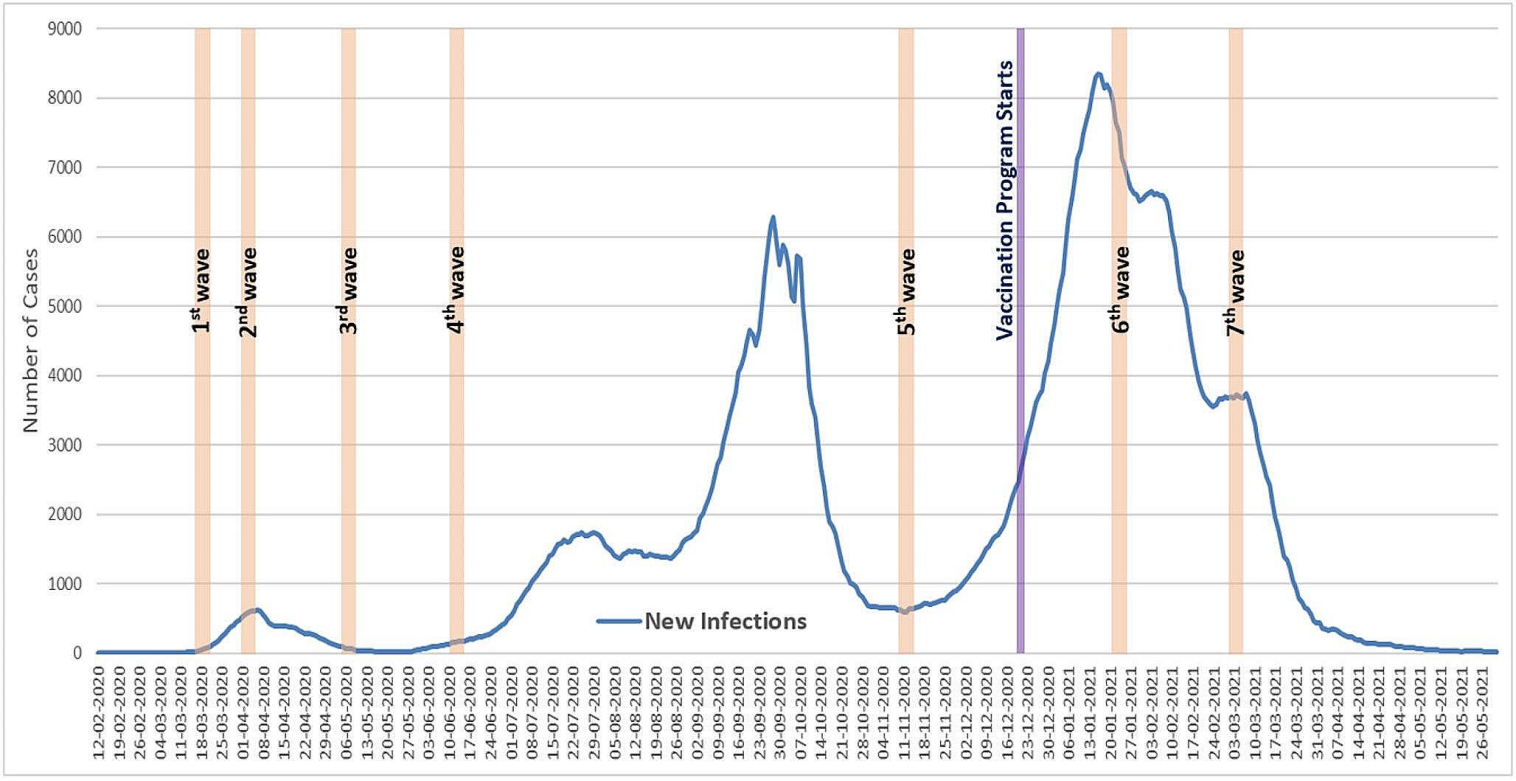
The seven-day moving average of new infections in Israel from February 2020 to May 2021. (Source: Israel’s Ministry of Health website; https://datadashboard.health.gov.il/COVID-19/general), plotted against the seven survey waves and the start of the vaccination program (December 20, 2020)

A large Israeli survey company experienced in conducting academic surveys over the Internet was contracted to run the survey. The survey was repeated seven times (hereafter referred to as the seven waves) from the outset of the spread of COVID-19 in Israel in March 2020 to March 2021 when the country was almost back to normal because of the availability of the vaccine. The goal was to have a panel of more than 1,000 respondents in all waves constituting a national and representative sample of the adult Jewish population in Israel. As a decline in the participation rate was expected, the first wave ended when 2,003 responses from participants 18 years or older had been received. The remaining surveys were distributed to all of the respondents of the first wave, and each wave ended after the goal of about 1,200 participants was reached.^1^ Thus, the study uses unbalanced panel data that includes seven data points. The survey waves are described in Table 1. Figure 1 depicts the timing of the seven waves in relation to the number of new infections each week from February 2020 to May 2021.

**Table 1:** Number of responses and the COVID-19 situation in Israel by wave

Wave number	Start date	Number of responses	Number of positive cases in Israel	COVID-19 situation in Israel
1	March 17, 2020	2,003	94	During lockdown, with high infection rate
2	April 2, 2020	1,201	719	During lockdown, with high infection rate
3	May 4, 2020	1,201	55	Infection rate is decreasing, and no lockdown
4	June 11, 2020	1,201	192	Infection rate is low, and no lockdown
5	November 11, 2020	1,202	765	Infection rate is increasing, and there are some restrictions but no lockdown. This wave administered 1.5 months before vaccination program starts
6	January 22, 2021	1,202	7,388	During lockdown, with high infection rate. Vaccination rate in population: First dose: 27.5%; second dose: 10.4%
7	March 2, 2021	1,202	4,378	Infection rate is decreasing, and no lockdown. Vaccination rate in population: First dose: 51.8%; second dose: 37.8%

### Questions and Definitions of the Variables

The survey questions were identical in all seven waves. Participants were asked about their emotions, cognitions regarding COVID-19, expectations regarding the spread of COVID-19 and the future economy of Israel, and their vaccination status. These variables were measured with the following methods.

#### Emotions

Participants were asked: “In the last two weeks, to what extent have you felt the emotions fear and anxiety?” Participants answered on a scale of 1 (*I have not felt the emotion the slightest bit*) to 5 (*I have felt the emotion more strongly than ever*). The variables *FEAR* and *ANXIETY* were defined by the answers.

#### Cognitions about COVID-19

Participants were asked: “Please estimate the probability (%) that you will be infected with COVID-19 within a month from now.” Participants were instructed to provide a value of 0–100%. The variable *PROB* was defined with the number. The survey also asked, “What do you expect your symptoms to be if you get infected?” Participants answered on a scale of 1 (*the symptoms would have little influence*) to 6 (*it would be extremely serious symptoms that could be fatal*). The variable *SEVERITY* was defined with the answer. Given that the subjective risk of suffering ill health from COVID-19 should depend on the multiplication of *PROB* by *SEVERITY*, the variable *HEALTH RISK* was defined as the interaction of the variables *PROB* and *SEVERITY*. For example, if a respondent believed that the probability of infection was zero, the subjective risk was zero, even if the person believed that the symptoms would be very serious if they were infected.

#### Expectations Regarding the Spread of COVID-19

Participants were asked to rate their expectations regarding the spread of COVID-19 in Israel in a month, from 1 (*the spread of the virus will completely end*) to 6 (*the virus will spread all over Israel and deaths will accumulate, completely paralyzing urban functions and the economy*). The variable *COVID SPREAD* was defined with the answer. In line with the emotion and cognition variables, lower values represent a better state or better expectations.

#### Expectations Regarding the Future Economy

The variables *OWN INCOME* and *ISRAEL GDP* represent the participants’ economic expectations about their income and Israel’s GDP in the year ahead, respectively.^2^ Participants were asked to choose an answer from 1 (*an increase of more than 4%*) to 6 (*a decrease of more than 10%*) indicating their expectations in both regards. Once again, lower values represent more optimistic expectations.

#### Participants’ Vaccination Status

As Pfizer’s COVID-19 vaccine became available in Israel in December 2020, participants were asked about getting vaccinated in Waves 6 and 7. They were asked to choose the applicable statement (1 = *I have received the first dose of the vaccine*; 2 = *I have received two doses of the vaccine*; 3 = *I am going to get the COVID-19 vaccine as soon as I can*; 4 = *I am going to get the COVID-19 vaccine when it becomes available to my age group*; or 5 = *I do not want to get the COVID-19 vaccine*). Three dummy variables were created: *1st SHOT* (1 if choosing 1 and 0 otherwise), *2nd SHOT* (1 if choosing 2 and 0 otherwise), and *AGAINST* (1 if choosing 5 and 0 otherwise), the last to control for vaccine hesitancy.

#### Additional Variables

Three additional variables were defined: (1) the cumulative number of vaccinated Israelis (*VACC RATE*)^3^ calculated as the number of vaccinated Israelis divided by Israel’s population, (2) the daily number of positive cases in Waves 1–4 (*NO. INFECTED 1to4*), and (3) the daily number of positive cases in Waves 5–7 (*NO. INFECTED 5to7*). Note, Waves 1 to 4 occurred during the first COVID-19 outbreak in Israel, in which the number of infected was about 700 cases per day. Waves 5 to 7 were during the third outbreak, in which the number of infected was more than 10 times higher at the peak. The magnitude of the effect of the number infected may be different for the two periods, so we adopted two variables: *NO. INFECTED 1to4*, which is the number infected in Waves 1 to 4, and 0 for the other waves, and *NO. INFECTED 5to7*, which is the number infected in Waves 5 to 7, and 0 for the other waves.

### Method of Estimation

The following fixed-effects model was estimated to examine the effects of a person’s vaccination status (*1st SHOT*, *2nd SHOT*) and the vaccination rate in Israel (*VACC RATE*):

$${Y_{it}} = {\rm{ }}{\alpha _0} + {\alpha _1}{1^{st}}SHO{T_{it}} + {\rm{ }}{\alpha _2}{2^{nd}}SHO{T_{it}} + {\rm{ }}{\alpha _3}VACC{\rm{ }}RAT{E_t}$$

$$ + {\alpha _4}NO.{\rm{ }}INFECTED{\rm{ }}1to{4_t} + {\rm{ }}{\alpha _5}NO.{\rm{ }}INFECTED\,5to{7_t}$$

1 $$ + \,{\alpha _6}Wave{\rm{ }}dummie{s_t}\, + \,{\alpha _7}AGAINS{T_{it}}\, + \,{k_i}\, + {u_{it}},$$

where *i* is an individual and *t* represents the wave of the survey. *Y*_*it*_ represents *HEALTH RISK*, *PROB*, *SEVERITY*, *FEAR*, *ANXIETY*, *COVID SPREAD*, *OWN INCOME*, and *ISRAEL GDP*, and *k*_*i*_ and *u*_*it*_ represent a fixed effect and a disturbance term, respectively. *HEALTH RISK* and *PROB* were estimated with a fixed-effects model because *PROB* is measured as a cardinal number, and subsequently, their coefficients were reported. However, since the other variables are ordered variables, they were estimated with the blow up and cluster (BUC) estimator [20], a fixed-effects ordered logit model, developed utilizing the conditional maximum likelihood estimator [21], which is available with the feologit command in Stata. We report the odds ratios, which are the exponentials of the estimates of the coefficients, and offer unequivocal *t*-test results. Dummies for the waves after Wave 5 are excluded because they were collinear with *VACC RATE*.

## Results

### Representativeness Check

First, the sample was checked for representativeness of the population of Israel. As Table S1A in Electronic Supplementary Material 1 indicates, the frequencies of the respondents and the national population stratified by gender and age groups revealed that the survey sample represented the national population quite well with respect to gender and age. The only exception was the lower rate of participation of those over age 65. To confirm that the results of this study (see “Main results”) represent the Israeli nation, Eq. 1 was estimated using these sampling weights of gender and age and revealed essentially the same results (see Electronic Supplementary Material 1, Tables S1B and S1C).

### Descriptive Statistics

**Fig. 2:**
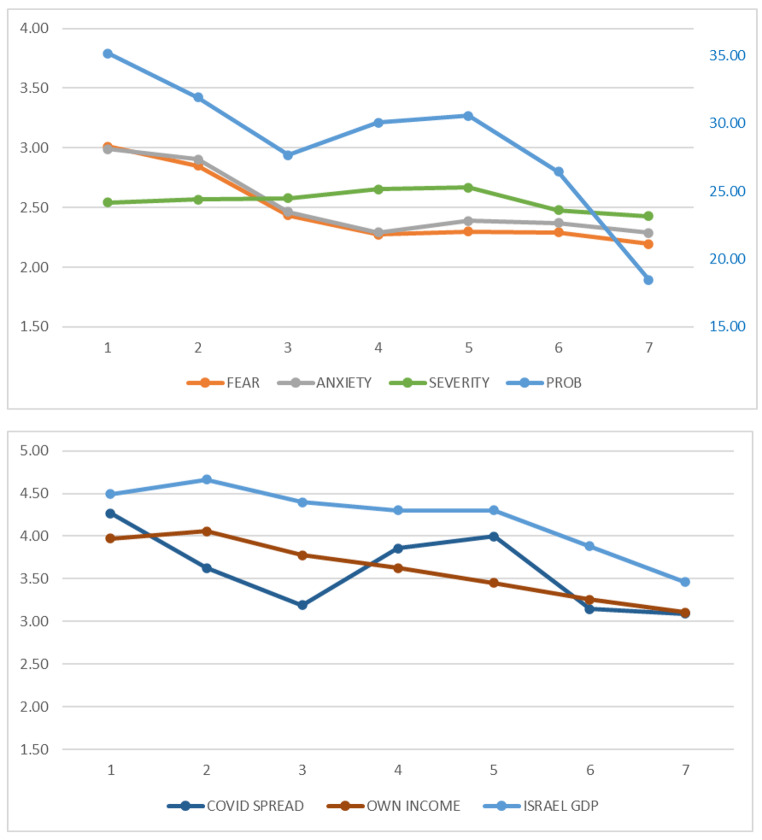
Means of the key variables of each survey. Top: Cognition and emotion variables. Bottom: Expectation variables. *FEAR* and *ANXIETY* = Fear and anxiety level experienced in the last two weeks, respectively (left *y* axis is the scale); *PROB* = probability of being infected with COVID-19 within a month, (right *y* axis is the scale); *SEVERITY* = severity of symptoms if infected; *COVID SPREAD* = expectations regarding the spread of COVID-19 in Israel in a month; *OWN INCOME* and *ISRAEL GDP* = economic expectations about participants’ income and Israel’s gross domestic product (GDP) in the year ahead, respectively

Table 2 presents the descriptive statistics of the eight dependent variables. To visualize the trends, we graphed the means in Fig. 2. Although the results indicate that the trends are not identical among the variables, there are some commonalities. First, the values in Wave 1 were relatively higher than those of other waves, and consequently most variables showed a downward trend over the observation period. Second, some variables, such as *PROB* and *COVID SPREAD*, show clear ups and downs, suggesting that the spread of COVID-19 and the lockdowns affected the participants’ cognitions and expectations about COVID-19. Third, many variables, such as *PROB*, *SEVERITY*, *COVID SPREAD*, and *ISRAEL GDP*, decreased in Wave 6. Since these dependent variables should change depending on various factors, this assumption is explored with a regression analysis controlling the fixed effects (see “Main results”).

**Table 2:** Descriptive statistics: Means and standard deviations (in parentheses) of main variables by wave

Variable	Wave						
	1	2	3	4	5	6	7
Cognition							
*PROB*	35.18	31.91	27.67	30.05	30.57	26.44	18.46
	(26.72)	(23.61)	(22.93)	(23.12)	(22.70)	(27.44)	(20.90)
*SEVERITY*	2.54	2.57	2.58	2.65	2.67	2.48	2.43
	(1.21)	(1.31)	(1.34)	(1.34)	(1.42)	(1.35)	(1.38)
*HEALTH RISK*	94.41	89.33	78.32	87.27	90.34	72.42	50.51
	(91.98)	(89.03)	(85.94)	(89.98)	(94.16)	(163.05)	(71.02)
Emotions							
*FEAR*	3.01	2.85	2.44	2.27	2.30	2.29	2.19
	(1.34)	(1.33)	(1.28)	(1.24)	(1.25)	(1.26)	(1.20)
*ANXIETY*	2.99	2.90	2.46	2.29	2.39	2.37	2.29
	(1.36)	(1.36)	(1.31)	(1.30)	(1.30)	(1.29)	(1.26)
Expectations							
*COVID SPREAD*	4.27	3.63	3.19	3.86	4.00	3.15	3.09
	(1.45)	(1.51)	(1.34)	(1.13)	(1.23)	(1.34)	(1.24)
*OWN INCOME*	3.97	4.06	3.78	3.63	3.45	3.26	3.10
	(1.40)	(1.39)	(1.24)	(1.25)	(1.32)	(1.25)	(1.08)
*ISRAEL GDP*	4.49	4.66	4.40	4.30	4.30	3.88	3.46
	(1.29)	(1.21)	(1.14)	(1.13)	(1.28)	(1.32)	(1.28)

Note: *PROB* = Probability of being infected with COVID-19 within a month; *SEVERITY* = severity of symptoms if infected; *HEALTH RISK* = the interaction of *PROB* and *SEVERITY*; *FEAR* and *ANXIETY* = fear and anxiety level experienced in the last two weeks, respectively; *COVID SPREAD* = expectations regarding the spread of COVID-19 in Israel in a month; *OWN INCOME* and *ISRAEL GDP* = economic expectations about participants’ income and Israel’s gross domestic product (GDP) in the year ahead, respectively

Table 3 lists the Kendall tau rank correlation coefficients between three vaccination variables, *1st SHOT*, *2nd SHOT*, and *VACC RATE*, and the dependent variables. All of the correlations were negative and significant except for that between *2nd SHOT* and *SEVERITY*, suggesting that the vaccination program improved the COVID-19 situation in Israel. A closer inspection reveals that the correlations with *2nd SHOT* were larger than those with *1st SHOT* except for *SEVERITY*. In addition, whereas the magnitude of the correlation of *VACC RATE* with the cognition and emotion variables was comparable to that of *2nd SHOT*, the correlations of *VACC RATE* with the expectation variables were larger than those of *2nd SHOT.*

**Table 3:** Correlation coefficients between key variables

Variable	1st SHOT	2nd SHOT	VACC RATE
Cognition			
*PROB*	-0.0211*	-0.1924***	-0.1626***
*SEVERITY*	-0.0243**	-0.0152	-0.0516***
*HEALTH RISK*	-0.0217*	-0.1632***	-0.1509***
Emotions			
*FEAR*	-0.0373***	-0.0985***	-0.1114***
*ANXIETY*	-0.0379***	-0.0818***	-0.0911***
Expectations			
*COVID SPREAD*	-0.1024***	-0.1271***	-0.1969***
*OWN INCOME*	-0.0808***	-0.1089***	-0.1864***
*ISRAEL GDP*	-0.0934***	-0.1481***	-0.23***

Note: *N* = 9,198 in all correlation pairs. *PROB* = Probability of being infected with COVID-19 within a month; *SEVERITY* = severity of symptoms if infected; *HEALTH RISK* = the interaction of *PROB* and *SEVERITY*; *FEAR* and *ANXIETY* = fear and anxiety level experienced in the last two weeks, respectively; *COVID SPREAD* = expectations regarding the spread of COVID-19 in Israel in a month; *OWN INCOME* and *ISRAEL GDP* = economic expectations about participants’ income and Israel’s gross domestic product (GDP) in the year ahead, respectively. *1st SHOT* and *2nd SHOT* = received first vaccine dose and received both vaccine doses, respectively; *VACC RATE* = number of vaccinated Israelis divided by Israel’s population. * *p* < .10. ** *p* < .05. *** *p* < .01

### Main Results: Estimates of a Fixed-effects Model

Table 4 presents the estimates of the model in Eq. 1. (Two alternative estimation methods to the BUC are shown in Electronic Supplementary Material 2, Tables S2A and S2B. The results are unchanged.) Whereas all estimates on *1st SHOT* were insignificant, those on *HEALTH RISK* and *PROB* had a negative sign and those on the other variables were less than unity. Negative signs in the fixed-effects model and odds ratios less than unity imply the same outcome: a negative association between *1st SHOT* and the dependent variables (a negative association implies that the dependent variable, such as *PROB* or *FEAR*, weakens when the person gets vaccinated), but one that is insignificant for all cases.

**Table 4:** Estimation results of Eq. 1: Baseline model including both *NO. INFECTED 1to4* and *NO. INFECTED 5to7*

Variable	(1)	(2)	(3)	(4)	(5)	(6)	(7)	(8)
	Coefficient by fixed-effects model		Odds ratio by fixed-effects ordered logit model					
	HEALTH RISK	PROB	SEVERITY	FEAR	ANXIETY	COVID SPREAD	OWN INCOME	ISRAEL GDP
*1st SHOT*	-1.688	-2.209	0.920	0.930	0.864	0.835	0.933	0.880
	(9.354)	(1.988)	(0.131)	(0.128)	(0.118)	(0.117)	(0.138)	(0.114)
*2nd SHOT*	-48.375***	-15.378***	0.660***	0.625***	0.607***	0.823	0.877	0.836
	(4.358)	(1.306)	(0.097)	(0.088)	(0.085)	(0.117)	(0.141)	(0.123)
*AGAINST*	-2.200	-1.228	0.927	0.816	0.999	1.747***	0.861	0.968
	(4.641)	(1.393)	(0.180)	(0.151)	(0.178)	(0.293)	(0.163)	(0.168)
*VACC RATE*	-7.328	-2.105	0.788	1.041	1.112	0.309***	0.477***	0.205***
	(5.249)	(1.435)	(0.121)	(0.157)	(0.167)	(0.044)	(0.077)	(0.031)
*NO. INFECTED 1to4*	0.100	0.099***	0.995**	1.045***	1.037***	1.011***	1.028***	1.009***
	(0.073)	(0.020)	(0.002)	(0.003)	(0.002)	(0.002)	(0.003)	(0.002)
*NO. INFECTED 5to7*	-0.001	0.001	0.9999*	1.000	1.000	0.9998***	1.000	1.000
	(0.001)	(0.001)	(0.001)	(0.001)	(0.001)	(0.001)	(0.001)	(0.001)
*wave2*	-74.197	-71.977***	48.414**	0.001***	0.001***	0.001***	0.001***	0.003***
	(52.254)	(14.544)	(78.779)	(0.001)	(0.001)	(0.001)	(0.001)	(0.004)
*wave3*	-16.430***	-8.504***	1.188	0.113***	0.154***	0.128***	0.450***	0.739***
	(3.618)	(1.021)	(0.130)	(0.013)	(0.017)	(0.014)	(0.050)	(0.077)
*wave4*	-27.181*	-24.650***	3.824***	0.001***	0.001***	0.058***	0.002***	0.113***
	(16.472)	(4.617)	(1.936)	(0.001)	(0.001)	(0.027)	(0.001)	(0.055)
Constant	90.312***	30.806***						
	(1.957)	(0.490)						
No. of observations	9,198	9,198	7,882	7,825	7,890	8,562	7,723	8,381
(Pseudo) *R*-squared	0.0516	0.0872	0.0121	0.1386	0.1139	0.1407	0.1225	0.1292
No. of individuals	2,005	2,005	1,327	1,334	1,334	1,456	1,313	1,422

Note: Robust standard errors are in parentheses and assume clustering at the individual level. Estimation method is the fixed-effects ordered logit model, except for *HEALTH RISK* and *PROB*, which are estimated with the fixed-effects model because *PROB* is measured as a cardinal number. Therefore, estimated coefficients are shown in Columns 1 and 2, and odds ratios are shown in Columns 3 to 8. The statistical significance is measured from 0 for coefficients and from 1 for odds ratios. The regression analysis does not include 14 participants from Wave 1 who did not answer the *PROB* question. The lower number of observations for the ordered fixed-effect estimations is due to the use of the blow up and cluster estimator [20], in which individuals who are observed only once or always answered with the same value over time are excluded by the program because their contribution to Chamberlain’s [21] conditional log likelihood, which is the objective function to be maximized, is zero [22, p. 267]. *PROB* = Probability of being infected with COVID-19 within a month; *SEVERITY* = severity of symptoms if infected; *HEALTH RISK* = the interaction of *PROB* and *SEVERITY*; *FEAR* and *ANXIETY* = fear and anxiety level experienced in the last two weeks, respectively; *COVID SPREAD* = expectations regarding the spread of COVID-19 in Israel in a month; *OWN INCOME* and *ISRAEL GDP* = economic expectations about participants’ income and Israel’s gross domestic product (GDP) in the year ahead, respectively. *1st SHOT* and *2nd SHOT* = received first vaccine dose and received both vaccine doses, respectively; *AGAINST* = no plans to get vaccinated; *VACC RATE* = number of vaccinated Israelis divided by Israel’s population, *NO. INFECTED 1to4* and *5to7* = daily number of positive cases in Waves 1–4 and 5–7, respectively. * *p* < .10. ** *p* < .05. *** *p* < .01

The estimates on *2nd SHOT* had a significant and negative association with the five cognition and emotion variables, whereas they were insignificant for the three expectation variables. In contrast, the estimates on *VACC RATE* were significantly and negatively associated with the three expectation variables, whereas they were insignificant for the cognition and emotion variables. The possible indirect effect of *VACC RATE* on *PROB* was insignificant. In sum, the estimates indicate that whereas most Israeli citizens understood the benefits to their personal safety of getting vaccinated, they did not realize that progress in the national vaccination rate should reduce their risk of getting infected.

Regarding the control variables, the odds ratios of *NO. INFECTED 1to4* for five variables (including *FEAR* and *ANXIETY* and expectations about the future economy) were significantly larger than unity, implying that the increase in the number of positive cases was reasonably associated with the deterioration in the participants’ emotions and expectations. In addition, the coefficient had a significant and positive sign for *PROB*. In sum, a larger *NO. INFECTED 1to4* was associated with larger values for six of the eight dependent variables. In contrast, *NO. INFECTED 5to7* was insignificant for seven of the eight dependent variables. In addition, in the significant case of *COVID SPREAD*, the odds ratio was smaller than unity, in contrast to what one would expect. These results suggest that the inclusion of this variable might be inappropriate.

Therefore, for the robustness check, the specification in which *NO. INFECTED 5to7* was excluded from Eq. 1 was estimated. The estimates were qualitatively the same as those in Table 4 (see Table S2C in Electronic Supplementary Material 2). Although the vaccination program did not start until after the fifth wave of the survey, we included the data from Waves 1–5. To be thorough, we estimated Eq. 1 without the data from Waves 1–4. Table S2D in Electronic Supplementary Material 2 shows the estimates using only the data from Waves 5–7. This timeframe covers the period after November 2020, which saw the third COVID-19 outbreak and the start of the vaccination program in Israel. Before the start of the vaccination program, *1st Shot* and *2nd Shot* have no variation as no one was vaccinated. This robustness check that excludes Waves 1 to 4 also refers to this issue, as only in the baseline (Wave 5) is there no variation for these variables. The results are similar to those in Table 4, confirming that the conclusions are robust to the selection of the observation period.

## Discussion

Using panel data from seven survey waves conducted from March 2020 to March 2021, we investigated how the vaccination against COVID-19 affected Israelis’ cognitions and emotions about the virus and their expectations about their future income and the national economy. Israel was the first country to embark on a rapid vaccination program. Thus, its citizens could not look to other countries to see the effectiveness of vaccination at the country level, making it a very suitable case study.

The descriptive statistics findings indicate that the values of most of the cognition, emotion, and expectation variables in the first wave were relatively higher than those of other waves, and consequently most variables showed a downward trend over the observation period. (In line with the emotion and cognition variables, lower values for the expectation variables represent a better state or better expectations.) This finding suggests that Israelis feared the unknown virus at the outset but habituated to it gradually. This phenomenon is called “pandemic fatigue.” Pandemic fatigue, defined by the World Health Organization [23] as “distress which can result in demotivation to follow recommended protective behaviors, emerging gradually over time and affected by a number of emotions, experiences, and perceptions,” has been identified as a risk factor for noncompliance with health-protective behavior by the public [24, 25]). With respect to the fourth COVID-19 outbreak in Israel, Bodas et al.’s [25] findings indicate that pandemic fatigue had cascading effects on vaccination efforts. In particular, during the fourth outbreak of the pandemic in Israel, threat perception components, such as concern and fear of contracting the disease, could not predict vaccination uptake. Instead, perception of the importance of the vaccine and its effectiveness were predictive of vaccination uptake.

Our descriptive statistics findings also suggest that some variables, such as *PROB* and *COVID SPREAD*, show clear ups and downs, suggesting that the spread of COVID-19 and the lockdowns affected the participants’ cognitions and expectations about COVID-19. Moreover, we found that *PROB*, *SEVERITY*, *COVID SPREAD*, and *ISRAEL GDP* decreased in Wave 6, even though this was the period with the highest number of infection cases in the population. One possible reason is the initiation of the vaccination program in December 2020, which led to the increase in the vaccination rate at that time and affected people’s expectations.

Our fixed-effects model findings indicate that the increase in the number of positive cases was reasonably associated with the deterioration in the participants’ emotions and expectations. In addition, our findings indicate that the estimates on *1st SHOT* were insignificant for the expectation variables and for the cognition variables, whereas the estimates on *2nd SHOT* had a significant and negative association with the five cognition and emotion variables. These results suggest that Israelis did not feel completely relieved until they had received the second dose. The results of the model also show that both getting vaccinated and the vaccination rate in Israel improved the respondents’ cognitions regarding COVID-19, their emotions, and their expectations about the future economy. In addition, we had hypothesized that getting vaccinated personally and the level of vaccination in the community would affect people through different paths. We found that although the participants’ vaccination status affected cognitions about their safety and their emotions, it did not affect their expectations regarding the situation in Israel as a whole. In contrast, although progress in the country’s vaccination rate affected expectations about the future, it did not affect the respondents’ cognitions regarding their safety or their emotions. To our knowledge, these results have not been reported elsewhere.

Our findings underscore the importance of getting vaccinated for emotional and cognitive relief on the individual level. They also show that high vaccination rates in the population improve people’s economic expectations, which is important for the recovery of the economy [18]. In fact, changes in people’s economic expectations about national economic growth as a result of vaccination rates are likely to promote changes in consumption and investment expenditures. These findings have important implications for the process of economic recovery from the pandemic and are helpful as a guide to the effects of vaccinations on stabilization policies.

## Electronic Supplementary Material

Below is the link to the electronic supplementary material.

Supplementary Material 1

## Data Availability

The datasets analysed during the current study are available from the corresponding author on reasonable request.

## Footnotes

1 Five additional participants were invited in Waves 3–7. When they were removed from the dataset, the main results were unchanged; see Electronic Supplementary Material 2, Table S2E.

2 The GDP growth rate in Israel was 4.46%, 4.38%, 3.98%, and 3.77% in 2016, 2017, 2018, and 2019, respectively.

3 The cumulative number of vaccinated Israelis is available on a designated website of Israel’s Ministry of Health. See https://datadashboard.health.gov.il/COVID-19/general. The daily number of positive cases is also available on the Ministry of Health’s website.
