# Supplementary material for: The Effect of Being Vaccinated and National Vaccination Rates on Individuals’ Cognitions, Emotions, and Economic Expectations: Evidence from Israel
Source: Int J Behav Med. 2024 Feb 12;32(5):788–97. doi: 10.1007/s12529-024-10269-3 (PMC12672598; doi:10.1007/s12529-024-10269-3)
Supplement: Supplementary file 1 — Supplementary Material 1 [file 12529_2024_10269_MOESM1_ESM.docx]

**Electronic Supplementary Material**

**The effect of being vaccinated and national vaccination rates on individuals' cognitions, emotions, and economic expectations: Evidence from Israel**

**1. REPRESENTATIVENESS OF THE DATA**

In Table S1A, we present the number of respondents and the national population stratified by gender and age group. The table reveals that the survey data represent the national population quite well with respect to gender and age except for fewer respondents age 65 or over.

Table S1A. Distribution of the respondents and Israeli population by gender and age

| Variable | Survey | | Population | |
| --- | --- | --- | --- | --- |
|  | Number | Ratio (%) | Number  (in thousands) | Ratio (%) |
| Men | 4,684 | 48.95 | 2,343 | 48.94 |
| Women | 4,518 | 51.05 | 2,444 | 51.06 |
| Total | 9,202 | 100 | 4,788 | 100 |
| Age group (years) |  |  |  |  |
| 15–29 | 2,479 | 0.29 | 1,338 | 0.28 |
| 30–39 | 2,449 | 0.27 | 864 | 0.18 |
| 40–49 | 1,660 | 0.17 | 769 | 0.16 |
| 50–64 | 2,090 | 0.22 | 914 | 0.19 |
| 65+ | 524 | 0.05 | 903 | 0.19 |
| Total | 9,202 | 100 | 4,788 | 100 |

Note: Survey age range: 18–79 years; population age range: 15–79 years. Data source: Israel’s Central Bureau of Statistics yearbook 2019. See [1].

The results suggest that the conclusions drawn from Table 4 in the main text apply to the Israeli population. To confirm this supposition, we estimated Equation 1 using the multiple sampling weights of gender and age. The estimates presented in Tables S1B and S1C are qualitatively the same as those in Table 4 of the main text. In Tables S1B and S1C, for each dependent variable, we show the estimates using sampling weights, followed by the original estimates copied from the main text. All the estimates of the weighted regressions were close to the unweighted ones, indicating that the main conclusions in this study apply to the representative samples of Israel.

Table S1B. Weighted estimations of Table 4 of the main text for the cognition variables and for *FEAR*

| Variable | (1) | (2) | (3) | (4) | (5) | (6) | (7) | (8) |
| --- | --- | --- | --- | --- | --- | --- | --- | --- |
|  | Coefficient  by fixed-effect model | | | | Odds ratio  by fixed-effect ordered logit model | | | |
|  | *HEALTH RISK*  weighted | *HEALTH RISK*  unweighted | *PROB*  weighted | *PROB*  unweighted | *SEVERITY*  weighted | *SEVERITY*  unweighted | *FEAR*  weighted | *FEAR*  unweighted |
| *1^st^ SHOT* | -2.796 | -1.688 | -2.562 | -2.209 | 0.925 | 0.920 | 1.013 | 0.930 |
|  | (7.805) | (9.354) | (1.799) | (1.988) | (0.147) | (0.131) | (0.156) | (0.128) |
| *2^nd^ SHOT* | -48.47*** | -48.375*** | -14.68*** | -15.378*** | 0.590*** | 0.660*** | 0.638*** | 0.625*** |
|  | (4.864) | (4.358) | (1.374) | (1.306) | (0.0958) | (0.097) | (0.102) | (0.088) |
| *AGAINST* | -2.078 | -2.200 | -1.044 | -1.228 | 0.951 | 0.927 | 0.807 | 0.816 |
|  | (4.537) | (4.641) | (1.433) | (1.393) | (0.187) | (0.180) | (0.150) | (0.151) |
| *VACC RATE* | -9.273* | -7.328 | -2.730* | -2.105 | 0.871 | 0.788 | 0.992 | 1.041 |
|  | (5.070) | (5.249) | (1.405) | (1.435) | (0.161) | (0.121) | (0.168) | (0.157) |
| *NO. INFECTED 1to4* | 0.0823 | 0.100 | 0.101*** | 0.099*** | 0.991*** | 0.995** | 1.045*** | 1.045*** |
|  | (0.0964) | (0.073) | (0.0233) | (0.020) | (0.00275) | (0.002) | (0.00278) | (0.003) |
| *NO. INFECTED 5to7* | -0.0007* | -0.001 | 0.001 | 0.001 | 0.9999*** | 0.9999* | 1.000 | 1.000 |
|  | (0.001) | (0.001) | (0.001) | (0.001) | (0.001) | (0.001) | (0.001) | (0.001) |
| *wave2* | -64.92 | -74.197 | -73.37*** | -71.977*** | 562.4*** | 48.414** | 0.001*** | 0.001*** |
|  | (68.88) | (52.254) | (16.74) | (14.544) | (1,110) | (78.779) | (0.001) | (0.001) |
| *wave3* | -18.99*** | -16.430*** | -9.252*** | -8.504*** | 1.416*** | 1.188 | 0.116*** | 0.113*** |
|  | (4.634) | (3.618) | (1.177) | (1.021) | (0.182) | (0.130) | (0.0152) | (0.013) |
| *wave4* | -25.34 | -27.181* | -25.27*** | -24.650*** | 7.706*** | 3.824*** | 0.001*** | 0.001*** |
|  | (21.86) | (16.472) | (5.343) | (4.617) | (4.730) | (1.936) | (0.001) | (0.001) |
| Constant | 92.38*** | 90.312*** | 30.33*** | 30.806*** |  |  |  |  |
|  | (2.447) | (1.957) | (0.540) | (0.490) |  |  |  |  |
| No. of observations | 9,187 | 9,198 | 9,187 | 9,198 | 7,882 | 7,882 | 7,817 | 7,825 |
| (Pseudo) *R*-squared | 0.065 | 0.052 | 0.100 | 0.087 | 0.0167 | 0.0121 | 0.1392 | 0.1386 |
| No. of individuals | 2,000 | 2,005 | 2,000 | 2,005 | 1,325 | 1,327 | 1,331 | 1,334 |

Note: Columns 2, 4, 6, and 8 are repeated from Table 4 in the main text for ease of comparison. Estimation method is the fixed-effect ordered logit model, except for *HEALTH RISK* and *PROB*, which are estimated with the fixed-effect model because *PROB* is measured as a cardinal number. Robust standard errors are in parentheses and assume clustering at the individual level. The statistical significance is measured from 0 for coefficients and from 1 for odds ratios. *PROB* = Probability of being infected with COVID-19 within a month; *SEVERITY* = severity of symptoms if infected; *HEALTH RISK* = the interaction of *PROB* and *SEVERITY*; *FEAR* = fear level experienced in the last two weeks; *1^st^ SHOT* and *2^nd^ SHOT* = received first vaccine dose and received both vaccine doses, respectively; *AGAINST* = no plans to get vaccinated; *VACC RATE* = number of vaccinated Israelis divided by Israel’s population, *NO. INFECTED 1to4* and *5to7* = daily number of positive cases in Waves 1–4 and 5–7, respectively. * *p* < .10. ** *p* < .05. *** *p* < .01.

Table S1C. Weighted estimations of Table 4 of the main text for *ANXIETY* and for the expectation variables

| Variable | (9) | (10) | (11) | (12) | (13) | (14) | (15) | (16) |
| --- | --- | --- | --- | --- | --- | --- | --- | --- |
|  | *ANXIETY*  weighted | *ANXIETY*  unweighted | *COVID SPREAD* weighted | *COVID SPREAD* unweighted | *OWN INCOME* weighted | *OWN INCOME* unweighted | *ISRAEL GDP* weighted | *ISRAEL GDP* unweighted |
| *1^st^ SHOT* | 0.886 | 0.864 | 0.855 | 0.835 | 0.995 | 0.933 | 0.963 | 0.880 |
|  | (0.127) | (0.118) | (0.136) | (0.117) | (0.157) | (0.138) | (0.130) | (0.114) |
| *2^nd^ SHOT* | 0.607*** | 0.607*** | 0.860 | 0.823 | 1.026 | 0.877 | 1.134 | 0.836 |
|  | (0.103) | (0.085) | (0.155) | (0.117) | (0.188) | (0.141) | (0.202) | (0.123) |
| *AGAINST* | 1.027 | 0.999 | 1.790*** | 1.747*** | 0.838 | 0.861 | 0.920 | 0.968 |
|  | (0.186) | (0.178) | (0.308) | (0.293) | (0.164) | (0.163) | (0.161) | (0.168) |
| *VACC RATE* | 1.025 | 1.112 | 0.341*** | 0.309*** | 0.440*** | 0.477*** | 0.161*** | 0.205*** |
|  | (0.179) | (0.167) | (0.0584) | (0.044) | (0.0828) | (0.077) | (0.0279) | (0.031) |
| *NO. INFECTED 1to4* | 1.036*** | 1.037*** | 1.011*** | 1.011*** | 1.026*** | 1.028*** | 1.007*** | 1.009*** |
|  | (0.00274) | (0.002) | (0.00228) | (0.002) | (0.00284) | (0.003) | (0.00250) | (0.002) |
| *NO. INFECTED 5to7* | 1.000 | 1.000 | 0.9998*** | 0.9998*** | 1.000 | 1.000 | 1.000 | 1.000 |
|  | (0.001) | (0.001) | (0.001) | (0.001) | (0.001) | (0.001) | (0.001) | (0.001) |
| *wave2* | 0.001*** | 0.001*** | 0.0001*** | 0.001*** | 0.001*** | 0.001*** | 0.0102** | 0.003*** |
|  | (0.001) | (0.001) | (0.001) | (0.001) | (0.001) | (0.001) | (0.0182) | (0.004) |
| *wave3* | 0.161*** | 0.154*** | 0.123*** | 0.128*** | 0.489*** | 0.450*** | 0.785* | 0.739*** |
|  | (0.0204) | (0.017) | (0.0161) | (0.014) | (0.0602) | (0.050) | (0.0989) | (0.077) |
| *wave4* | 0.00017*** | 0.000*** | 0.0468*** | 0.058*** | 0.00303*** | 0.002*** | 0.169*** | 0.113*** |
|  | (0.000101) | (0.000) | (0.0238) | (0.027) | (0.00187) | (0.001) | (0.0938) | (0.055) |
| No. of observations | 7,882 | 7,890 | 8,554 | 8,562 | 7,715 | 7,723 | 8,377 | 8,381 |
| (Pseudo) *R*-squared | 0.1165 | 0.1139 | 0.1358 | 0.1407 | 0.1136 | 0.1225 | 0.1185 | 0.1292 |
| No. of individuals | 1,341 | 1,344 | 1,453 | 1,456 | 1,310 | 1,331 | 1,421 | 1,422 |

Note: Columns 10, 12, 14, and 16 are repeated from Table 4 in the main text for ease of comparison. Estimation method is the fixed-effect ordered logit model, and odds ratios are shown. Robust standard errors are in parentheses and assume clustering at the individual level. *ANXIETY* = anxiety level experienced in the last two weeks; *COVID SPREAD* = expectations regarding the spread of COVID-19 in Israel in a month; *OWN* *INCOME* and *ISRAEL GDP* = economic expectations for participants’ own income and Israel’s gross domestic product (GDP) in the year ahead, respectively; *1^st^ SHOT* and *2^nd^ SHOT* = received first vaccine dose and received both vaccine doses, respectively; *AGAINST* = no plans to get vaccinated; *VACC RATE* = number of vaccinated Israelis divided by Israel’s population, *NO. INFECTED 1to4* and *5to7* = daily number of positive cases in Waves 1–4 and 5–7, respectively. * *p* < .10. ** *p* < .05. *** *p* < .01.

**2.** **ADDITIONAL INFORMATION ON THE ESTIMATES OF THE BASELINE MODEL**

Tables S2A and S2B show the results of a robustness check of the main results using the BUC-τ and random-effects ordered logit model (REOLM) methods. BUC and BUC-τ are the estimation method of fixed-effects ordered logistic models (see [2-4]). REOLM and BUC-τ are similar to BUC but use a different sampling method to estimate fixed effects and thresholds of an ordered variable separately.

Table S2A. Estimation results of Equation 1, using the blow up and cluster (BUC), BUC-τ, and random-effects ordered logit model (REOLM) estimation methods for *SEVERITY*, *FEAR*, and *ANXIETY*

| Variable | *SEVERITY* | | | *FEAR* | | | *ANXIETY* | | |
| --- | --- | --- | --- | --- | --- | --- | --- | --- | --- |
|  | BUC | BUC- τ | REOLM | BUC | BUC- τ | REOLM | BUC | BUC- τ | REOLM |
| *1^st^ SHOT* | 0.920 | 0.914 | 0.906 | 0.930 | 0.981 | 0.945 | 0.864 | 0.949 | 0.827 |
|  | (0.131) | (0.135) | (0.113) | (0.128) | (0.137) | (0.118) | (0.118) | (0.131) | (0.104) |
| *2^nd^ SHOT* | 0.660*** | 0.647*** | 0.741** | 0.625*** | 0.687*** | 0.625*** | 0.607*** | 0.665*** | 0.624*** |
|  | (0.097) | (0.100) | (0.101) | (0.088) | (0.097) | (0.079) | (0.085) | (0.093) | (0.078) |
| *AGAINST* | 0.927 | 0.922 | 0.870 | 0.816 | 0.816 | 0.749 | 0.999 | 1.043 | 0.899 |
|  | (0.180) | (0.184) | (0.143) | (0.151) | (0.164) | (0.132) | (0.178) | (0.198) | (0.156) |
| *VACC RATE* | 0.788 | 0.801 | 0.750** | 1.041 | 1.001 | 1.044 | 1.112 | 1.052 | 1.109 |
|  | (0.121) | (0.129) | (0.105) | (0.157) | (0.155) | (0.143) | (0.167) | (0.160) | (0.150) |
| *NO. INFECTED 1to4* | 0.995** | 0.994*** | 0.996* | 1.045*** | 1.044*** | 1.044*** | 1.037*** | 1.035*** | 1.037*** |
|  | (0.002) | (0.002) | (0.002) | (0.003) | (0.003) | (0.002) | (0.002) | (0.003) | (0.002) |
| *NO. INFECTED 5to7* | 1.000* | 1.000* | 1.000* | 1.000 | 1.000 | 1.000 | 1.000 | 1.000 | 1.000 |
|  | (0.001) | (0.001) | (0.001) | (0.001) | (0.001) | (0.001) | (0.001) | (0.001) | (0.001) |
| *wave2* | 48.414** | 68.649** | 17.596* | 0.001*** | 0.001*** | 0.001*** | 0.001*** | 0.001*** | 0.001*** |
|  | (78.779) | (113.154) | (26.340) | (0.001) | (0.001) | (0.001) | (0.001) | (0.001) | (0.001) |
| *wave3* | 1.188 | 1.177 | 1.104 | 0.113*** | 0.119*** | 0.120*** | 0.154*** | 0.163*** | 0.155*** |
|  | (0.130) | (0.131) | (0.106) | (0.013) | (0.014) | (0.012) | (0.017) | (0.019) | (0.016) |
| *wave4* | 3.824*** | 4.286*** | 2.705** | 0.001*** | 0.001*** | 0.001*** | 0.001*** | 0.001*** | 0.001*** |
|  | (1.936) | (2.196) | (1.253) | (0.001) | (0.001) | (0.001) | (0.001) | (0.001) | (0.001) |
| No. of observations | 7882 | 8142 | 9,213 | 7825 | 7923 | 9,213 | 7890 | 7968 | 9,213 |
| (Pseudo) *R*-squared | 0.012 | 0.496 |  | 0.139 | 0.433 |  | 0.11394 | 0.407 |  |
| No. of individuals | 1327 | 1388 | 2,008 | 1334 | 1365 | 2,008 | 1344 | 1369 | 2,008 |

Note: The BUC columns repeat the estimates from Table 4 in the main text to ease the comparison of the estimates by the different methods. Robust standard errors are in parentheses and assume clustering at the individual level. *SEVERITY* = severity of symptoms if infected; *FEAR* and *ANXIETY* = fear and anxiety level experienced in the last two weeks, respectively; *1^st^ SHOT* and *2^nd^ SHOT* = received first vaccine dose and received both vaccine doses, respectively; *AGAINST* = no plans to get vaccinated; *VACC RATE* = number of vaccinated Israelis divided by Israel’s population, *NO. INFECTED 1to4* and *5to7* = daily number of positive cases in Waves 1–4 and 5–7, respectively. **p* < .10. ***p* < .05. ****p* < .01.

Table S2B. Estimation results of Equation 1, using the blow up and cluster (BUC), BUC-τ, and random-effects ordered logit model (REOLM) estimation methods for *COVID SPREAD*, *OWN INCOME*, and *ISRAEL GDP*

| Variables | *COVID SPREAD* | | | *OWN INCOME* | | | *ISRAEL GDP* | | |
| --- | --- | --- | --- | --- | --- | --- | --- | --- | --- |
|  | BUC | BUC- τ | REOLM | BUC | BUC- τ | REOLM | BUC | BUC- τ | REOLM |
| *1^st^ SHOT* | 0.835 | 0.862 | 0.829 | 0.933 | 0.880 | 0.956 | 0.880 | 0.867 | 0.947 |
|  | (0.117) | (0.119) | (0.097) | (0.138) | (0.129) | (0.110) | (0.114) | (0.111) | (0.104) |
| *2^nd^ SHOT* | 0.823 | 0.818 | 0.860 | 0.877 | 0.968 | 1.046 | 0.836 | 0.804 | 0.954 |
|  | (0.117) | (0.114) | (0.099) | (0.141) | (0.148) | (0.124) | (0.123) | (0.118) | (0.115) |
| *AGAINST* | 1.747*** | 1.662*** | 1.475** | 0.861 | 0.793 | 0.811 | 0.968 | 0.889 | 0.894 |
|  | (0.293) | (0.281) | (0.227) | (0.163) | (0.157) | (0.127) | (0.168) | (0.163) | (0.135) |
| *VACC RATE* | 0.309*** | 0.320*** | 0.330*** | 0.477*** | 0.447*** | 0.491*** | 0.205*** | 0.235*** | 0.210*** |
|  | (0.044) | (0.046) | (0.039) | (0.077) | (0.071) | (0.061) | (0.031) | (0.036) | (0.026) |
| *NO. INFECTED 1to4* | 1.011*** | 1.010*** | 1.014*** | 1.028*** | 1.025*** | 1.026*** | 1.009*** | 1.008*** | 1.009*** |
|  | (0.002) | (0.002) | (0.002) | (0.003) | (0.003) | (0.002) | (0.002) | (0.002) | (0.002) |
| *NO. INFECTED 5to7* | 1.000*** | 1.000*** | 1.000*** | 1.000 | 1.000 | 1.000 | 1.000 | 1.000 | 1.000 |
|  | (0.001) | (0.001) | (0.001) | (0.001) | (0.001) | (0.001) | (0.001) | (0.001) | (0.001) |
| *wave2* | 0.001*** | 0.001*** | 0.001*** | 0.001*** | 0.001*** | 0.001*** | 0.003*** | 0.004*** | 0.002*** |
|  | (0.001) | (0.001) | (0.001) | (0.001) | (0.001) | (0.001) | (0.004) | (0.007) | (0.003) |
| *wave3* | 0.128*** | 0.134*** | 0.119*** | 0.450*** | 0.460*** | 0.457*** | 0.739*** | 0.766** | 0.679*** |
|  | (0.014) | (0.016) | (0.012) | (0.050) | (0.051) | (0.043) | (0.077) | (0.080) | (0.061) |
| *wave4* | 0.058*** | 0.067*** | 0.029*** | 0.002*** | 0.004*** | 0.003*** | 0.113*** | 0.136*** | 0.106*** |
|  | (0.027) | (0.031) | (0.012) | (0.001) | (0.002) | (0.001) | (0.055) | (0.068) | (0.045) |
| No. of observations | 8562 | 8667 | 9,213 | 7723 | 8568 | 9,213 | 8381 | 8584 | 9,213 |
| (Pseudo) *R*-squared | 0.1407 | 0.4775 |  | 0.1225 | 0.5433 |  | 0.1292 | 0.4854 |  |
| No. of individuals | 1456 | 1488 | 2,008 | 1313 | 1471 | 2,008 | 1422 | 1471 | 2,008 |

Note: The BUC columns repeat the estimates from Table 4 in the main text to ease the comparison of the estimates by the different methods. Robust standard errors are in parentheses and assume clustering at the individual level. *COVID SPREAD* = expectations regarding the spread of COVID-19 in Israel in a month; *OWN* *INCOME* and *ISRAEL GDP* = economic expectations for participants’ own income and Israel’s gross domestic product (GDP) in the year ahead, respectively; *1^st^ SHOT* and *2^nd^ SHOT* = received first vaccine dose and received both vaccine doses, respectively; *AGAINST* = no plans to get vaccinated; *VACC RATE* = number of vaccinated Israelis divided by Israel’s population, *NO. INFECTED 1to4* and *5to7* = daily number of positive cases in Waves 1–4 and 5–7, respectively. **p* < .10. ***p* < .05. ****p* < .01.

Table S2C shows the results of a robustness check that excludes *NO. INFECTED 5to7* from the estimation of Equation 1. Table S2D shows the results of a robustness check that excludes the data of Waves 1–4 from the estimation of Equation 1.

Table S2C. Estimation results of Equation 1, excluding *NO. INFECTED 5to7*

| Variable | (1) | (2) | (3) | (4) | (5) | (6) | (7) | (8) |
| --- | --- | --- | --- | --- | --- | --- | --- | --- |
|  | Coefficient  by fixed-effect model | | Odds ratio  by fixed-effect ordered logit model | | | | | |
|  | *HEALTH RISK* | *PROB* | *SEVERITY* | *FEAR* | *ANXIETY* | *COVID SPREAD* | *OWN INCOME* | *ISRAEL GDP* |
| *1^st^ SHOT* | -4.262 | -1.917 | 0.826 | 0.989 | 0.877 | 0.468*** | 0.884 | 0.815* |
|  | (10.724) | (2.188) | (0.105) | (0.125) | (0.108) | (0.059) | (0.123) | (0.098) |
| *2^nd^ SHOT* | -49.595*** | -15.240*** | 0.624*** | 0.641*** | 0.611*** | 0.645*** | 0.858 | 0.807 |
|  | (4.638) | (1.317) | (0.089) | (0.090) | (0.085) | (0.087) | (0.137) | (0.119) |
| *AGAINST* | -5.274 | -0.879 | 0.816 | 0.885 | 1.019 | 0.861 | 0.806 | 0.885 |
|  | (4.316) | (1.260) | (0.142) | (0.148) | (0.162) | (0.134) | (0.136) | (0.140) |
| *VACC RATE* | -7.132 | -2.127 | 0.798 | 1.041 | 1.112 | 0.311*** | 0.477*** | 0.207*** |
|  | (5.323) | (1.443) | (0.122) | (0.158) | (0.167) | (0.043) | (0.077) | (0.031) |
| *NO. INFECTED 1to4* | 0.131* | 0.096*** | 0.996*** | 1.045*** | 1.037*** | 1.018*** | 1.028*** | 1.010*** |
|  | (0.070) | (0.020) | (0.002) | (0.002) | (0.002) | (0.002) | (0.003) | (0.002) |
| Constant | 89.120*** | 30.945*** |  |  |  |  |  |  |
|  | (2.493) | (0.417) |  |  |  |  |  |  |
| No. of observations | 9,198 | 9,198 | 7,882 | 7,825 | 7,890 | 8,562 | 7,723 | 8,381 |
| (Pseudo) *R*-squared | 0.0510 | 0.0871 | 0.0221 | 0.1385 | 0.1139 | 0.1139 | 0.1224 | 0.1290 |
| No. of individuals | 2,005 | 2,005 | 1,327 | 1,334 | 1,334 | 1,456 | 1,313 | 1,422 |

Note: Estimation method is the fixed-effect ordered logit model, except for *HEALTH RISK* and *PROB*, which are estimated with the fixed-effect model because *PROB* is measured as a cardinal number. Though Waves 2–4 dummies are included for estimation, they are not shown in this table to save space. Robust standard errors are in parentheses and assume clustering at the individual level. The statistical significance is measured from 0 for coefficients and from 1 for odds ratios. *PROB* = Probability of being infected with COVID-19 within a month; *SEVERITY* = severity of symptoms if infected; *HEALTH RISK* = the interaction of *PROB* and *SEVERITY*; *FEAR* and *ANXIETY* = fear and anxiety level experienced in the last two weeks, respectively; *COVID SPREAD* = expectations regarding the spread of COVID-19 in Israel in a month; *OWN* *INCOME* and *ISRAEL GDP* = economic expectations for participants’ own income and Israel’s gross domestic product (GDP) in the year ahead, respectively; *1^st^ SHOT* and *2^nd^ SHOT* = received first vaccine dose and received both vaccine doses, respectively; *AGAINST* = no plans to get vaccinated; *VACC RATE* = number of vaccinated Israelis divided by Israel’s population, *NO. INFECTED 1to4* = daily number of positive cases in Waves 1–4.

**p* < .10. ***p* < .05. ****p* < .01.

Table S2D. Estimation results of Equation 1: Waves 5, 6, and 7 only

| Variable | (1) | (2) | (3) | (4) | (5) | (6) | (7) | (8) |
| --- | --- | --- | --- | --- | --- | --- | --- | --- |
|  | Coefficient  by fixed-effect model | | Odds ratio  by fixed-effect ordered logit model | | | | | |
|  | *HEALTH RISK* | *PROB* | *SEVERITY* | *FEAR* | *ANXIETY* | *COVID SPREAD* | *OWN INCOME* | *ISRAEL GDP* |
| *1^st^ SHOT* | -5.172 | -3.276 | 0.829 | 0.934 | 0.901 | 0.678** | 1.030 | 0.844 |
|  | (9.544) | (2.036) | (0.138) | (0.156) | (0.151) | (0.112) | (0.176) | (0.132) |
| *2^nd^ SHOT* | -52.978*** | -16.621*** | 0.488*** | 0.624*** | 0.676** | 0.581*** | 0.928 | 0.717* |
|  | (4.794) | (1.325) | (0.088) | (0.111) | (0.119) | (0.104) | (0.187) | (0.133) |
| *AGAINST* | -1.760 | -2.142 | 1.059 | 0.714 | 1.059 | 2.220*** | 1.092 | 0.942 |
|  | (4.824) | (1.323) | (0.244) | (0.157) | (0.234) | (0.481) | (0.274) | (0.210) |
| *VACC RATE* | -3.218 | -0.869 | 0.978 | 1.027 | 0.955 | 0.367*** | 0.396*** | 0.192*** |
|  | (5.257) | (1.423) | (0.183) | (0.191) | (0.175) | (0.063) | (0.080) | (0.037) |
| *NO. INFECTED 5to7* | -0.000 | 0.000 | 1.000 | 1.000 | 1.000 | 0.9998*** | 0.9999* | 1.000 |
|  | (0.000) | (0.000) | (0.001) | (0.001) | (0.001) | (0.001) | (0.001) | (0.001) |
| Constant | 90.139*** | 30.455*** |  |  |  |  |  |  |
|  | (2.185) | (0.499) |  |  |  |  |  |  |
| No. of observations | 3,606 | 3,606 | 2,417 | 2,237 | 2,222 | 2,946 | 2,012 | 2,797 |
| (Pseudo) *R*-squared | 0.0615 | 0.1114 | 0.0406 | 0.0139 | 0.0112 | 0.2386 | 0.0652 | 0.2036 |
| No. of individuals | 1,339 | 1,339 | 840 | 778 | 775 | 1,023 | 703 | 976 |

Note: Estimation method is the fixed-effect ordered logit model, except for *HEALTH RISK* and *PROB*, which are estimated with the fixed-effect model because *PROB* is measured as a cardinal number. Robust standard errors are in parentheses and assume clustering at the individual level. The statistical significance is measured from 0 for coefficients and from 1 for odds ratios. *PROB* = Probability of being infected with COVID-19 within a month; *SEVERITY* = severity of symptoms if infected; *HEALTH RISK* = the interaction of *PROB* and *SEVERITY*; *FEAR* and *ANXIETY* = fear and anxiety level experienced in the last two weeks, respectively; *COVID SPREAD* = expectations regarding the spread of COVID-19 in Israel in a month; *OWN* *INCOME* and *ISRAEL GDP* = economic expectations for participants’ own income and Israel’s gross domestic product (GDP) in the year ahead, respectively; *1^st^ SHOT* and *2^nd^ SHOT* = received first vaccine dose and received both vaccine doses, respectively; *AGAINST* = no plans to get vaccinated; *VACC RATE* = number of vaccinated Israelis divided by Israel’s population, *NO. INFECTED 5to7* = daily number of positive cases in Waves 5–7. **p* < .10. ***p* < .05. ****p* < .01.

Table S2E shows the results of a robustness check when the five participants who did not participate in Wave 1 are excluded.

Table S2E. Estimation results of Equation 1, excluding the five participants who did not participate in Wave 1

| Variable | (1) | (2) | (3) | (4) | (5) | (6) | (7) | (8) |
| --- | --- | --- | --- | --- | --- | --- | --- | --- |
|  | Coefficient  by fixed-effect model | | Odds ratio  by fixed-effect ordered logit model | | | | | |
|  | *HEALTH RISK* | *PROB* | *SEVERITY* | *FEAR* | *ANXIETY* | *COVID SPREAD* | *OWN INCOME* | *ISRAEL GDP* |
| *1^st^ SHOT* | -2.242 | -1.764 | 0.920 | 0.927 | 0.856 | 0.833 | 0.933 | 0.880 |
|  | (1.988) | (9.353) | (0.131) | (0.128) | (0.117) | (0.116) | (0.138) | (0.114) |
| *2^nd^ SHOT* | -15.422*** | -48.523*** | 0.661*** | 0.628*** | 0.603*** | 0.821 | 0.873 | 0.832 |
|  | (1.308) | (4.365) | (0.097) | (0.088) | (0.084) | (0.117) | (0.140) | (0.122) |
| *AGAINST* | -1.335 | -2.380 | 0.923 | 0.806 | 0.995 | 1.742*** | 0.867 | 0.969 |
|  | (1.393) | (4.648) | (0.180) | (0.150) | (0.177) | (0.293) | (0.164) | (0.168) |
| *VACC RATE* | -2.078 | -7.257 | 0.788 | 1.036 | 1.108 | 0.309*** | 0.479*** | 0.205*** |
|  | (1.436) | (5.255) | (0.121) | (0.157) | (0.166) | (0.044) | (0.078) | (0.031) |
| *NO. INFECTED 1to4* | 0.099*** | 0.099 | 0.995** | 1.045*** | 1.037*** | 1.011*** | 1.028*** | 1.009*** |
|  | (0.020) | (0.073) | (0.002) | (0.003) | (0.002) | (0.002) | (0.003) | (0.002) |
| *NO. INFECTED 5to7* | 0.001 | -0.001 | 1.000* | 1.000 | 1.000 | 1.000*** | 1.000 | 1.000 |
|  | (0.001) | (0.001) | (0.001) | (0.001) | (0.001) | (0.001) | (0.001) | (0.001) |
| Constant | 30.832*** | 90.395*** |  |  |  |  |  |  |
|  | (0.490) | (1.959) |  |  |  |  |  |  |
| No. of observations | 9,187 | 9,187 | 7,878 | 7,817 | 7,882 | 8,554 | 7,715 | 8,377 |
| (Pseudo) *R*-squared | 0.087 | 0.052 | 0.012 | 0.1387 | 0.1141 | 0.1406 | 0.1225 | 0.1294 |
| No. of individuals | 2,000 | 2,000 | 1,325 | 1,331 | 1,341 | 1,453 | 1,310 | 1,421 |

Note: Estimation method is the fixed-effect ordered logit model, except for *HEALTH RISK* and *PROB*, which are estimated with the fixed-effect model because *PROB* is measured as a cardinal number. Though Waves 2–4 dummies are included for estimation, they are not shown in this table to save space. Robust standard errors are in parentheses and assume clustering at the individual level. The statistical significance is measured from 0 for coefficients and from 1 for odds ratios. *PROB* = Probability of being infected with COVID-19 within a month; *SEVERITY* = severity of symptoms if infected; *HEALTH RISK* = the interaction of *PROB* and *SEVERITY*; *FEAR* and *ANXIETY* = fear and anxiety level experienced in the last two weeks, respectively; *COVID SPREAD* = expectations regarding the spread of COVID-19 in Israel in a month; *OWN* *INCOME* and *ISRAEL GDP* = economic expectations for participants’ own income and Israel’s gross domestic product (GDP) in the year ahead, respectively; *1^st^ SHOT* and *2^nd^ SHOT* = received first vaccine dose and received both vaccine doses, respectively; *AGAINST* = no plans to get vaccinated; *VACC RATE* = number of vaccinated Israelis divided by Israel’s population, *NO. INFECTED 1to4* and *5to7* = daily number of positive cases in Waves 1–4 and 5–7, respectively. **p* < .10. ***p* < .05. ****p* < .01.

**References**

1. Israel’s Central Bureau of Statistics yearbook 2019. Available at: https://www.cbs.gov.il/en/publications/Pages/2019/Statistical-Abstract-of-Israel-2019-No-70.aspx

2. Baetschmann G. Identification and estimation of thresholds in the fixed effects ordered logit model. *Econ Lett.* 2012;115:416-418.

3. Baetschmann G, Ballantyne A, Staub KE, Winkelmann R. feologit: A new command for fitting fixed-effects ordered logit models. *Stata J.* 2020;20:253-275.

4. Baetschmann G, Staub KE, Winkelmann R. Consistent estimation of the fixed effects ordered logit model. *J R Stat Soc Ser A.* 2015;178:685-703. <https://doi.org/10.1111/rssa.12090>.
